# Classification across gene expression microarray studies

**DOI:** 10.1186/1471-2105-10-453

**Published:** 2009-12-30

**Authors:** Andreas Buness, Markus Ruschhaupt, Ruprecht Kuner, Achim Tresch

**Affiliations:** 1 German Cancer Research Center (DKFZ), Department of Molecular Genome Analysis, 69120 Heidelberg, Germany; 2 Institute of Medical Informatics, Biometrics, and Epidemiology (IBE), LMU, 81377 Munich, Germany; 3 Gene Center Munich and Center for integrated Protein Science CiPSM, Department of Chemistry and Biochemistry, Ludwig-Maximilians-Universität München, 81377 Munich, Germany

## Abstract

**Background:**

The increasing number of gene expression microarray studies represents an important resource in biomedical research. As a result, gene expression based diagnosis has entered clinical practice for patient stratification in breast cancer. However, the integration and combined analysis of microarray studies remains still a challenge. We assessed the potential benefit of data integration on the classification accuracy and systematically evaluated the generalization performance of selected methods on four breast cancer studies comprising almost 1000 independent samples. To this end, we introduced an evaluation framework which aims to establish good statistical practice and a graphical way to monitor differences. The classification goal was to correctly predict estrogen receptor status (negative/positive) and histological grade (low/high) of each tumor sample in an independent study which was not used for the training. For the classification we chose support vector machines (SVM), predictive analysis of microarrays (PAM), random forest (RF) and k-top scoring pairs (kTSP). Guided by considerations relevant for classification across studies we developed a generalization of kTSP which we evaluated in addition. Our derived version (DV) aims to improve the robustness of the intrinsic invariance of kTSP with respect to technologies and preprocessing.

**Results:**

For each individual study the generalization error was benchmarked via complete cross-validation and was found to be similar for all classification methods. The misclassification rates were substantially higher in classification across studies, when each single study was used as an independent test set while all remaining studies were combined for the training of the classifier. However, with increasing number of independent microarray studies used in the training, the overall classification performance improved. DV performed better than the average and showed slightly less variance. In particular, the better predictive results of DV in across platform classification indicate higher robustness of the classifier when trained on single channel data and applied to gene expression ratios.

**Conclusions:**

We present a systematic evaluation of strategies for the integration of independent microarray studies in a classification task. Our findings in across studies classification may guide further research aiming on the construction of more robust and reliable methods for stratification and diagnosis in clinical practice.

## Background

Transcriptional profiling studies of human diseases aim to identify causal molecular mechanisms as well as to improve diagnosis. For example, in breast cancer several prognostic gene signatures have been proposed [1-4]. To this date, one has been approved for clinical diagnosis. Despite this success, molecular signatures based on microarray gene expression data may be unstable and thus still need to be considered with caution [5]. Similarly, the lack of agreement between different signatures raises doubts about the reliability and robustness of reported predictive gene lists [6]. Insufficient sample sizes, heterogeneity of tumor samples and patient characteristics are common obstacles concerning microarray data analysis. The integration of multiple studies may overcome this limitation. However, different protocols and technologies hamper such attempts and the translation to clinical practice. This particularly affects predictive signatures derived from gene expression microarray data. For example, a drop in predictive accuracy across two different technology platforms measuring a common set of samples has been found [7]. The misclassification rate raised from 2 to 19.5% in this study. On the other hand promising classification results for the integration of studies were reported [8] as well as a high level of concordance between several microarray-based and alternative technology platforms measuring gene expression [9].

**Figure 1:**
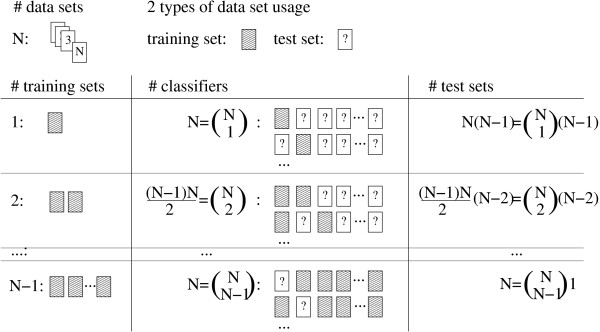
**Schematic overview of the systematic approach to assess the classification performance across independent data sets**. The role of each data set is exhaustively alternated between training and testing. *N *equals 4 in case of the estrogen receptor status and equals 3 in case of the histological grade, see Table 1.

Here, we establish a systematic approach to assess the performance of the integration of independent gene expression microarray data sets for classification across studies (Figure 1), propose a tailored classification method for this purpose and evaluate several methods on four independent human breast cancer studies comprising almost 1000 tumor samples.

### Preliminaries

#### Data integration and classification

The limited sample size in microarray studies always raises the question whether the sample under investigation is representative of the population. This is a major concern for any prediction task, and therefore it is highly desireable to consider all available data from related studies. Still then, it is unclear whether a meta-analysis increases the performance of a classifier, since technical and experimental settings vary between studies and introduce an additional layer of variation which might undo the benefits of a larger sample size. Therefore, any arbitrary integration of available data may not be the best choice. Nonetheless, an adequate strategy for the integration of heterogenous data sets may ideally help to identify those signals or genes which are not only most relevant for the classification task but also least variable with respect to the underlying experimental and technical differences. This helps to construct more robust gene signatures and ultimately leads to classifiers of increased predictive power.

#### Gene expression microarrays

Experimental and technical differences of microarray gene expression studies hamper data integration. Gene expression microarray platforms are based upon the principle of hybridization. The most important difference between platforms are the sequences used to measure the mRNA of a given gene. Not only the length of the sequence varies but also the choice of the most representative sequence for any particular gene. Moreover, the choice of the sequence itself influences the intensity of the signal independent of the given mRNA abundance. For example, the number of physical positions which can bind a fluorescent dye varies. Besides that, the total of all sequences on the microarray as well as the experimental processing influence the cross-hybridization properties. Thus, it is not only difficult to decide which measurements respectively sequences correspond to the same mRNA, but even in the case of matching sequences the measurement characteristics still vary. For example, the offset, scale, dynamic range and behavior as well as the level of noise differs. Another level of complexity arises from the use of two-channel/two-color platforms and single channel platforms. The two-channel platforms mostly quantify all measurements relative to a common reference sample. As a consequence the offset of the measurement is completely dependent on the choice of the reference. Nonetheless, the influence of the reference approximately cancels out if one focuses on differences in mRNA abundance between classes. Here, we disregard all issues of sequence matching including exons, introns, splicing variants etc. and rely solely on the gene annotation of probes or probe sets. The gene symbol serves as basis for the comparison of annotated probes across platforms. Note that any analysis will be hampered by different, technology-specific probe affinities for the same gene, by measurement failures as well as by wrongly annotated probes.

#### Classification across studies

The major issue when focusing on the experimental and technical aspect of classification across studies can be summarized as follows: different gene expression profiling platforms or studies measure the expression of the same common gene with different precision and on a different scale. Nonetheless, a common way to represent the gene expression measurements does not only allow to directly combine microarray data sets, but also to readily apply the generated classifier on a new data set which is represented in the same manner. To this end, [10,11] proposed the method TSP (top scoring pair) and [12] the generalized version kTSP (k-top scoring pairs), classifiers which directly refer to the relative ranks, i.e. the ordering of the actual gene expression values within a profile. kTSP was shown to perform as good as state-of-the-art algorithms while using a relatively small number of genes for classification. In addition, enhanced types of this approach have been developed and successfully applied to integrate gene expression studies for classification [13,14]. Independently, [8] introduced several variants to represent microarray data based upon the relative ranks of the gene expression values. Standard classification techniques were applied to binned or scaled quantile discretized data. Here, we choose the same general approach based upon the relative ranks and focus on the quantile transformed gene expression values. This approach appears to be the most simple and intuitive. In particular, it is relatively insensitive to preprocessing, e.g. scaling and normalization.

## Results

We systematically evaluated the generalization performance of five selected methods SVM, PAM, RF, kTSP and DV on four breast cancer gene expression microarray studies almost comprising 1000 independent samples (Table 1). The challenge was to predict estrogen receptor status (negative/positive) and histological grade (low/high) of a tumor sample in an independent study which was not used for the training. The prediction of estrogen receptor status based on gene expression data can be considered to be an achievable task. It has already been proposed to be presumably more accurate than standard clinical procedures [15]. The reliability of histological grade is questioned when used for the staging of tumors. Attempts to correlate histological grade with gene expression measurements suggest to refine the assigned grading status of the tumor sample [16]. We decided to focus on the two extreme gradings G1 (low) and G3 (high) while obtaining the highest contrast which is presumably most reliable and informative. It should be mentioned that estrogen receptor status and grading are associated in the complete data set. Low grade tumors have a strong tendency to be estrogen receptor status positive or from a different viewpoint estrogen receptor status negative tumors have a strong tendency to be of high grade. However, the prediction of estrogen receptor status itself does not suffice to predict histological grade since the estrogen status positive samples are fairly balanced between low and high grade (66% and 44%, respectively) in contrast to estrogen status negative samples (8% and 92%, respectively).

**Table 1:** Four breast cancer gene expression profiling studies

	Study (Reference)	Main Focus	Platform	Samples	ER-	ER+	G1	G3
1	[1]	wound-response signature	oligo (ratios)	295	69	226	75	119
2	[2]	p53 status signature	U133a/U133b	247	34	213	64	54
3	[16]	grade and prognosis	U133a	104	24	74	29	36
4	[4]	metastasis signature	U133a	286	77	209	-	-

**Figure 2:**
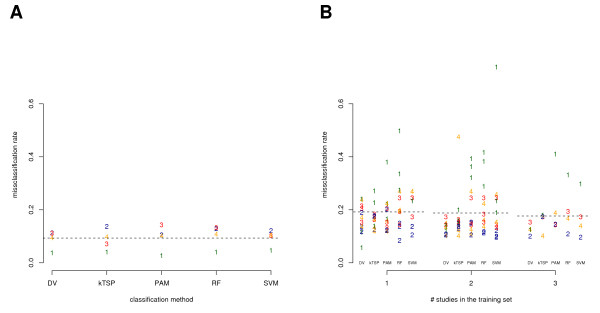
**The misclassification error for each of the five classification methods and each of the studies (*N *= 4) is shown for the estrogen receptor status**. The plotted numbers in distinct colors indicate the study as listed in Table 1 while pointing to the corresponding misclassification rate. A: The misclassification rate was estimated with complete cross-validation in each study separately. B: The misclassification rate is shown for each training set combination subgrouped by the number of studies used in the training (see Figure 1). Dotted lines indicate averages across classification methods.

**Figure 3:**
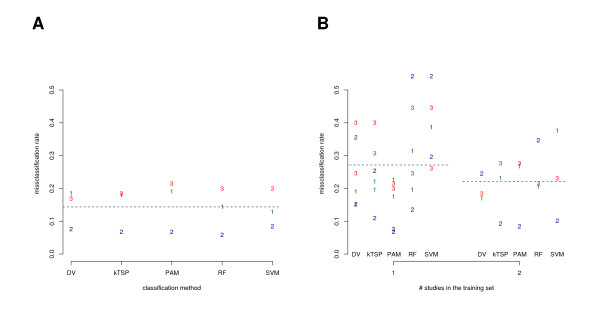
**The misclassification error for each of the five classification methods and each of the studies (*N *= 3) is shown for histological grade**. The plotted numbers in distinct colors indicate the study as listed in Table 1 while pointing to the corresponding misclassification rate. A: The misclassification rate was estimated with complete cross-validation in each study separately. B: The misclassification rate is shown for each training set combination subgrouped by the number of studies used in the training (see Figure 1). Dotted lines indicate averages across classification methods.

First of all, we benchmarked the studies while using a complete cross-validation approach to estimate the misclassification rate. This was done separately for each study, classification method and task (Figures 2A, 3A). Overall we found a good prediction accuracy. The average misclassification rate was 9% (14%) for estrogen receptor status (histological grade). All five classification methods performed comparably well (see Additional File 1). To systematically assess classification across studies and platforms and the potential benefit of data integration we applied our general approach as outlined in Figure 1. As major result we obtained a distinct drop of almost 10% in accuracy for classification across studies compared to the benchmark result of the cross-validation. The average misclassification rate was found to be 18% (22%) for estrogen receptor status (histological grade) measured on an independent study which was not used for the training. These results refer to the best case in which all remaining studies were combined for the training of the classifier (Figures 2B, 3B and Additional File 2). A weak decrease of the misclassification rate with increasing number of studies used in the training set was observed. This trend generally supports the notion to integrate studies for classification, but the benefit is marginal in this particular analysis. The misclassification rate found in study 1 was highly variable in case of the estrogen receptor status (Figure 2B). This observation coincides with the fact that study 1 differs in its technology platform from all other studies. Gene expression ratios instead of intensities were measured in study 1 (Table 1). Our proposed method DV shows better prediction accuracy on study 1 than all other methods in almost all training set compositions (Figures 2B, 3B). As intended, the method appears to be particularly robust in classification across platforms. This observations mainly accounts for the better performance than average (13% and 20% compared to 18% and 22%) and for comparably less variance in performance (see Additional File 2). Similar results were obtained for variations on the parameter *F *and confirm our findings based on DV with *F *= 4 (*F *= 2: 14% and 20%, and *F *optimized between 2 or 6: 15% and 18%; data not shown).

**Figure 4:**
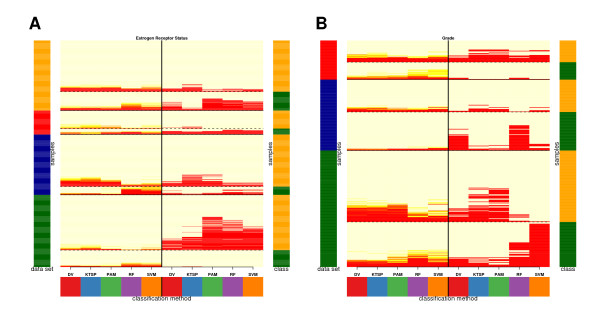
**The figure summarizes the main classification results while detailing class, data set and sample specific prediction performance (A: estrogen receptor status in four studies; B: histological grade in three studies)**. Samples correspond to rows and methods to columns. The estimates of the cross-validation approach are shown on the left separated by a vertical line from the results of the classification across studies on the right where the number of training sets was maximal (A:3, B:2). The graphical representation is similar to a heatmap. The area corresponding to a misclassified sample is labelled in red and in light yellow for a correctly classified sample. The error estimates of the repeated cross-validation have been mapped to the range from red to light yellow for each individual sample. The cross-validation approach was run separately for each study. For the classification across studies the results are shown in which all studies except the one used for assessment formed the training set (see Additional File 3 and 4 for the results of all training set combinations). Samples are ordered by study, class, their average misclassification rate in the cross-validation and classification across studies. The color code at the left indicates the study (green = 1, blue = 2, red = 3, orange = 4), at the right the class (A: green = ER-, orange = ER+; B: green = G1, orange = G3).

The major classification results are visualized in Figure 4 comprising the outcome of the complete cross-validation approach applied to each study separately and of the classification across studies which were obtained when the training set was maximal. A complete graphical representation of all classification results can be found in the Additional Files 3 and 4. The visualization offers a way to unravel systematic differences. The prediction result is depicted per individual sample. Correctly and falsely classified samples can be monitored across the methods. Misclassified samples are marked in red. The results are ordered with respect to class membership, study, classification method and approach. This complex representation allows to identify samples exhibiting characteristic behaviour. Interestingly to note, there appear to be red cluster or stripes which relate to samples with a consistent tendency in failing classification regardless of the method and approach. Moreover, the border between the two classes shifts but the affected samples which in turn fail classification are rather the same across the methods. The combined results help to identify 'marginal' samples in each class which are most distant to the core of the class and are thus prone to misclassification and secondly, to identify samples which persist any correct classification.

### Gene signatures

The final classifier for estrogen receptor status which we obtained when integrating all four independent studies comprises 50 genes for the methods DV and kTSP (see Additional File 5). This gene list includes the three genes *FOXA1, GATA3 *and *SLC39A6 *which have been previously reported in a ten gene signature [15]. The ten gene signature was found to be highly predictive for estrogen receptor status. Our final classifier of PAM is based upon 115 genes (see Additional File 5). Again three genes *FOXA1, GATA3 *and *ESR1 *overlap with the ten gene signature. All three final classifiers contain several genes which are well known to be estrogen responders like *AGR2, STC2, TFF1 *or *XBP1 *. These findings confirm the relevance and predictivity of the gene signatures. The classifiers obtained by the methods SVM and RV contain 1000 genes since no further feature selection was applied. The final classifier for histological grade upon the DV and kTSP method was derived after the integration of three studies which contain grading annotation (Table 1). This classifier is based on the following 20 genes: *APOC1, CENPA, CKS2, CXCL10, FST, GJA1, H2AFZ, HMGB3, HN1, KIF13B, KIF2C, NAT1, NOVA1, PCM1, PNRC2, QDPR, SCUBE2, SEC61G, STC2, UBE2C *. This signature contains genes which are strongly linked to breast cancer progression in previous studies. *APOC1 *has been identified in a multiprotein index upon postoperative serum proteomic profiles which is associated to metastatic relapse in high-risk breast cancer patients receiving adjuvant chemotherapy [17]. Protein expression of *STC2 *correlates with longer disease free survival [18]. *KIF2C *is overexpressed in breast cancer cells and functional analysis suggest a link between overexpression and carcinogenesis [19]. RNAi based inhibition of *KIF2C *expression inhibits growth in breast cancer cell lines. Furthermore, reactivation of the potential tumor suppressor connexin *GJA1 (Cx43) *leads to reduced cell migration and regulated various angiogenesis linked proteins in breast cancer cell lines [20]. These findings indicate that the signature is not only predictive for histological grade of breast cancer specimen, but can also be linked to tumor progression. Thus, the signature may contain further important candidate genes even though predictive signatures must not be related to any causal molecular mechanism in general. The 60 genes of PAM classifier strongly overlap with the previous list of 20 genes (see Additional File 5). 10 genes are common to both lists: *CENPA, GJA1, HMGB3, KIF13B, KIF2C, NAT1, QDPR, SCUBE2, STC2, UBE2C*. This concordance between the signatures for histological grade across different methods confirms their relevance. Moreover, the observed partial overlap of the predictive signatures derived for estrogen receptor status and grade may relate to the aforementioned association of estrogen receptor status negative tumors and high grade. For example, in case of DV and kTSP the intersection of the corresponding signatures comprises the three genes *NAT1, STC2 *and *SCUBE2 *.

## Discussion

The complete cross-validation procedure separately applied to each microarray study and classification method gives an unbiased estimate and benchmark for the misclassification rate. The rather large sample sizes of the studies tend to control the variance of this estimate. Thus, the distinct drop of accuracy in classification across studies (Figures 2 and 3) has to be assigned to methodological shortcomings, underlying experimental differences and other factors which distinguish the studies and sample collectives. We present an approach to systematically evaluate and quantify the effect of data integration on the accuracy in across studies classification. A weak, but consistent decrease of the misclassification rate with increasing number of studies used in the training set was observed. At least the potential shortcomings of the computational methods used for the classification across studies remain a not very well studied area in research to this date. Our framework allows to assess any computational method for data integration and classification across studies and our proposed method DV shows favorable characteristics which together may guide further research.

The drastic increase of the dimensionality when considering pairs of genes instead of genes adds to the complexity of the problem. However, the good performance of kTSP and DV may indicate an interesting characteristic of gene expression microarray data which account for their success despite the dimensionality. Recent results highlight the importance of the dependence structure in microarray gene expression data and discuss the advantage of focusing on differences of ordered, non-overlapping pairs of log-transformed gene expression values [21]. This adds evidence that a well chosen step into the space of pairs or possibly even triples of genes does not only increase the dimensionality and associated difficulties, but may allow to focus on the relevant structure present in the data.

Our straightforward extension of the kTSP method evaluated in the setting of classification across studies and platforms performed comparably well. Nonetheless, further improvements of DV can be anticipated. The impact on the classification rule when failing to measure a single gene varies for the different genes. The loss of information on a single gene affect all related gene pairs which are not necessarily equally distributed between the genes. To further balance the importance of the genes in the majority vote across all gene pairs, one may introduce a two stage voting scheme. For each gene the majority vote of all pairs which contain the gene is determined and then in a second step the majority of all resulting individual gene votes defines the prediction.

Moreover, it is worthwhile to mention that the simple counting type nature of the score Δ_*ij *_does allow to integrate any further microarray data set easily in an already existing classifier rule and hence does allow to extend the sample size. However, the properties of such a classifier require further validation steps. Moreover, when aiming to build a reasonable classifier one should recall the counting nature of the score Δ_*ij *_and ensure a minimal number samples of possibly at least 20 samples in total. Besides that, the preselection of the genes which is based upon the kTSP method itself might be replaced by a more tailored strategy accounting for the interconnected classification rule of DV. Additionally, faster alternatives like a feature reduction which preselects all gene pairs with the highest variance in their relative difference may be evaluated. Moreover, other promising variants of kTSP remain to be analyzed. For example, instead of building the classifier upon the relation between pairs of genes, one might consider relations between features like set of genes, summarized networks or pathways. This can include mixtures like the matrix of all relations between genes and summarized networks.

The consistent misclassification of a core of samples independently of the method and training set composition indirectly confirms generalization power. These misclassifications might be caused by technical or experimental issues of the mRNA processing and measurement or by wrong sample annotations and constraints at the time of the class assignment in the clinic. Improved methods in clinical practice which profit from the feedback and findings made by gene expression microarray studies may overcome such limitations in future. For example, quantitative RT-PCR or robust low-density array platforms may become a more important alternative to existing clinical procedures. Moreover, the identification of 'marginal' samples as well as sample which persistently failed correct classification may be helpful to rebuild and refine the classifier which then need to be validated in subsequent studies.

Normalization or calibration procedures often rely on the assumption of no change in expression of housekeeping genes or overall genes. This assumption may be of critical importance in diagnostic testing which is based on low-density arrays or quantitative RT-PCR where only a small number of genes is measured. Thus, classification methods solely relying on the relative quantification between pairs or sets of genes like kTSP and DV appear to be especially well suited. Despite their simplicity they show competitive performance and may play an important role in future clinical tests.

## Conclusions

The integration and combined analysis of gene expression microarray studies remains still a challenge. Mainly, different technologies hamper the integration and translation to clinical practice. We propose a tailored classification method and a systematic approach to unbiasedly assess the benefit of integration of independent studies. This approach aims to establish good statistical practice. We analyzed four human transcriptomic breast cancer studies on different platforms comprising almost 1000 samples, evaluated five classification methods and exemplary derived classifiers for the prediction of estrogen receptor status and histological grade. In summary, our proposed method performed favorable. Particularly, it showed superior performance in the across platform classification setting when trained on single channel data. Our results will guide further research aiming on more reliable diagnostic and prognostic gene signatures in clinical practice.

## Methods

### Data Sets

Four independent and publicly available breast cancer microarray studies were compiled and prepared for the analysis as described elsewhere [22]. We restricted ourselves to those 9765 unique Entrez gene identifiers which were common to all data sets. In addition, 85 samples in study [16] already present in study [2] were removed prior to the analysis and only tumor samples with documented histological grade (377) or estrogen receptor status (926) were included in the analysis (Table 1).

### Generalization of kTSP

The kTSP (k-top scoring pairs) method is a binary classifier which is composed of *k *elementary classifiers. Each elementary classifier is based upon a pair of genes of 'high predictive power'. This means that gene *i *has a prevalently higher expression value than gene *j *in one class and vice versa in the other class. An unknown sample is then classified according to the relative expression values of gene *i *and *j *in that sample. The kTSP method simply collects the votes from the *k *best disjoint elementary classifiers and reports the majority vote as its decision. Each gene is allowed to participate in only one elementary classifier, because this makes the decision rule robust against systematic errors in the measurements of a single gene. On the other hand, an "indicator" gene that is very high in one group and very low in the other is very likely to be part of many high performance elementary classifiers. In our derived version (DV) of kTSP, we tradeoff between robustness and performance by introducing an upper bound *F *that limits the number of classifiers that contain a common gene. Our derived version selects gene pairs by starting with the highest scoring gene pair and then successively adjoining the next highest scoring gene pair for which none of its two constituents is already contained in *F *previously selected gene pairs. Note that the parameter choice *F *= 1 results in the original kTSP method when only considering all pairs of genes in the set of two times *k *genes which have been selected by the kTSP classifier.

More formally, let the measurements be given as a genes × samples matrix (*q*_*gs*_), where *q*_*gs *_denote the relative rank of the expression value of gene *g *in sample *s *among all expression values of sample *s *(i.e. *q*_*gs *_is the rank of gene *g *in sample *s*, divided by the number of genes in sample *s *for which measurements are avaliable). Let *c*_*s *_∈ {± 1} be the class of sample *s*. Let *n*_*c *_be the number of samples in class *c*. The essential building block compares the expression values of two genes *i, j *and is given by the score |Δ _*ij*_|,

If we had to build an elementary classifier for a new sample s*based on the relative expression of gene *i *and *j *only, the Bayes classifier would be

and the Bayes error would be decreasing for increasing score |Δ _*ij*_|, see [12].

The final decision of DV is again made by majority vote. An extensive and more algorithmic description of DV is given in the supplements (see Additional File 6).

### Analysis

All analysis was done using R [23] and Bioconductor [24]. We selected the following five methods for the analysis: support vector machines (SVM) with radial basis kernel, predictive analysis of microarrays (PAM), random forest (RF), kTSP and our generalization DV [12,25-27]. The parameter *k *of kTSP which controls the number of non-overlapping gene pairs was optimized between the choices 10, 25 and 50. In case of DV the kTSP method was used as a feature reduction technique and *k *was optimized for the same choices. *F *of DV was set to 4.

To estimate the misclassification rate for each individual data set via complete cross-validation we used the package MCRestimate [28]. The whole process of a (outer) 5-fold cross-validation was repeated ten times. Parameter were optimized in a nested (inner) 5-fold cross-validation. The normalized data sets constrained to the 9765 common unique Entrez gene identifiers were used without any further preprocessing as described elsewhere [22]. A preprocessing step was applied in each cross-validation step to reduce the number of features. The 1000 genes with highest variance were selected.

Our approach to evaluate classification across studies is outlined in Figure 1. Each study is either used as training or test set in a systematic manner. This covers all possible combinations and assures an unbiased estimate of the generalization power. As in the complete cross-validation approach 1000 genes were selected in a preprocessing step. Those 1000 genes were chosen which were found to have the highest variance across all samples in the training set after quantile normalization to the mean gene expression of the corresponding pooled quantiles. After gene selection, PAM, SVM and RF were directly trained and tested on the original quantiles i.e. on the data for each sample which resulted from the replacement of the gene expression value by its quantile prior to the gene selection. The same preprocessing for gene selection was used for kTSP and DV but the methods were then applied to the original normalized gene expression values.

## Authors' contributions

AB, AT and MR developed the methodology. AB performed the analysis and drafted the manuscript. AB and MR implemented the algorithms. AB and RK analysed the signatures. All authors participated in the design of the study, contributed to the manuscript and approved the final version.

## Supplementary Material

Additional file 1 **Average misclassification rate**. The average misclassification rate for each of the five classification methods is shown as bar (A: estrogen receptor status; B: histological grade). The misclassification rate was estimated with cross-validation in each study separately. The average and the standard deviation across the four studies is visualized. Click here for file

Additional file 2 **Average misclassification rate across studies**. The figure displays the average misclassification rate on an independent breast cancer study for five classification methods. (A: estrogen receptor status; B: histological grade). The average is calculated across the different studies. The misclassification rate for each study itself is the average rate of all classifiers in which the study was not used in the training. The results are shown separately with respect to the number of contributing studies which formed the training set. The dotted lines indicate averages across all classification methods and visualize a tendency of a decreasing error rate with an increasing number of studies which were used for the training of the classifier. Click here for file

Additional file 3 **Classification results across studies for estrogen receptor status**. The figure summarizes all classification results for estrogen receptor status. Samples correspond to rows and methods to columns. The estimates of the cross-validation approach are shown on the left separated by a vertical line from the results of the classification across studies on the right. For the latter the samples of the studies used for the training are marked in gray and the ones not used are shown in white. The cross-validation approach was run separately for each study. Misclassified samples are labelled in red and correctly classified ones in light yellow. The error estimates of the repeated cross-validation have been mapped to the range from red to light yellow. Samples are ordered by study, class, their average misclassification rate in the cross-validation and classification across studies. The color code at the bottom indicates the method (red = DV, blue = kTSP, green = PAM, purple = RF, orange = SVM), at the left the study (green = 1, blue = 2, red = 3, orange = 4), at the right the class (green = ER-, orange = ER+). Click here for file

Additional file 4 **Classification results across studies for histological grade**. The figure summarizes all classification results for the histological grade. Samples correspond to rows and methods to columns. The estimates of the cross-validation approach are shown on the left separated by a vertical line from the results of the classification across studies on the right. For the latter the samples of the studies used for the training are marked in gray and the ones not used are shown in white. The cross-validation approach was run separately for each study. Misclassified samples are labelled in red and correctly classified ones in light yellow. The error estimates of the repeated cross-validation have been mapped to the range from red to light yellow. Samples are ordered by study, class, their average misclassification rate in the cross-validation and classification across studies. The color code at the bottom indicates the method (red = DV, blue = kTSP, green = PAM, purple = RF, orange = SVM), at the left the study (green = 1, blue = 2, red = 3), at the right the histological grade (green = G1, orange = G3). Study 4 is not included since the histological grade of the samples was not available. Click here for file

Additional file 5 **Gene signatures**. Gene signatures for the prediction of estrogen receptor status and histological grade derived from the complete data set. Click here for file

Additional file 6 **Description of DV**. Algorithmic description of DV. Click here for file
