# Supplementary figures and images for: Classification across gene expression microarray studies
Source: BMC Bioinformatics. 2009 Dec 30;10:453. doi: 10.1186/1471-2105-10-453 (PMC2811711; doi:10.1186/1471-2105-10-453)

**A**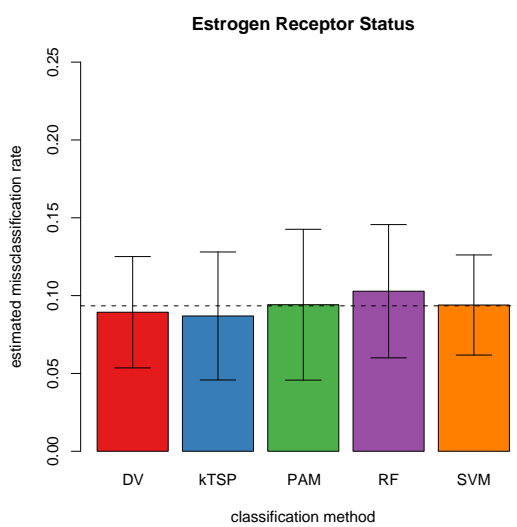**B**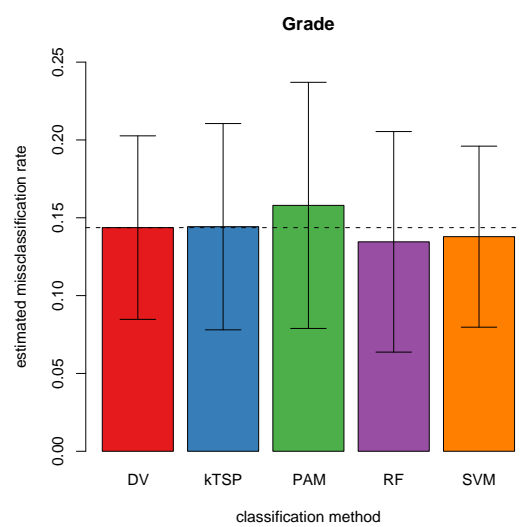

Supplement: Additional file 1 — Average misclassification rate. The average misclassification rate for each of the five classification methods is shown as bar (A: estrogen receptor status; B: histological grade). The misclassification rate was estimated with cross-validation in each study separately. The average and the standard deviation across the four studies is visualized. [file 1471-2105-10-453-S1.PDF]

**A**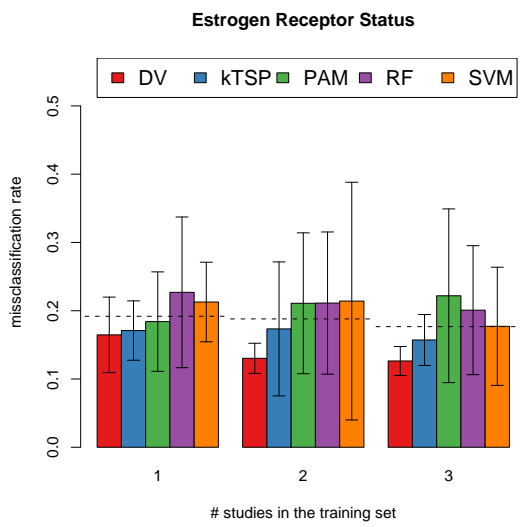**B**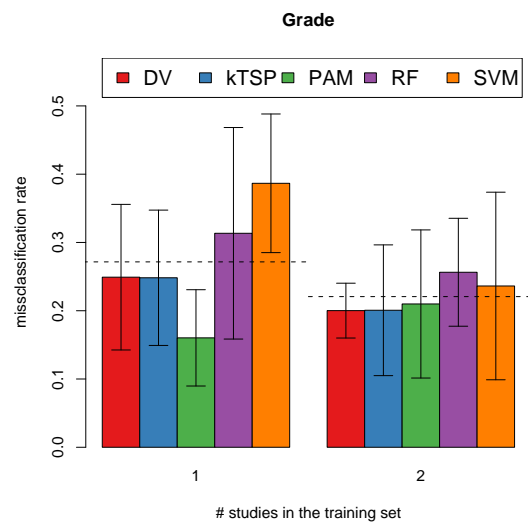

Supplement: Additional file 2 — Average misclassification rate across studies. The figure displays the average misclassification rate on an independent breast cancer study for five classification methods. (A: estrogen receptor status; B: histological grade). The average is calculated across the different studies. The misclassification rate for each study itself is the average rate of all classifiers in which the study was not used in the training. The results are shown separately with respect to the number of contributing studies which formed the training set. The dotted lines indicate averages across all classification methods and visualize a tendency of a decreasing error rate with an increasing number of studies which were used for the training of the classifier. [file 1471-2105-10-453-S2.PDF]

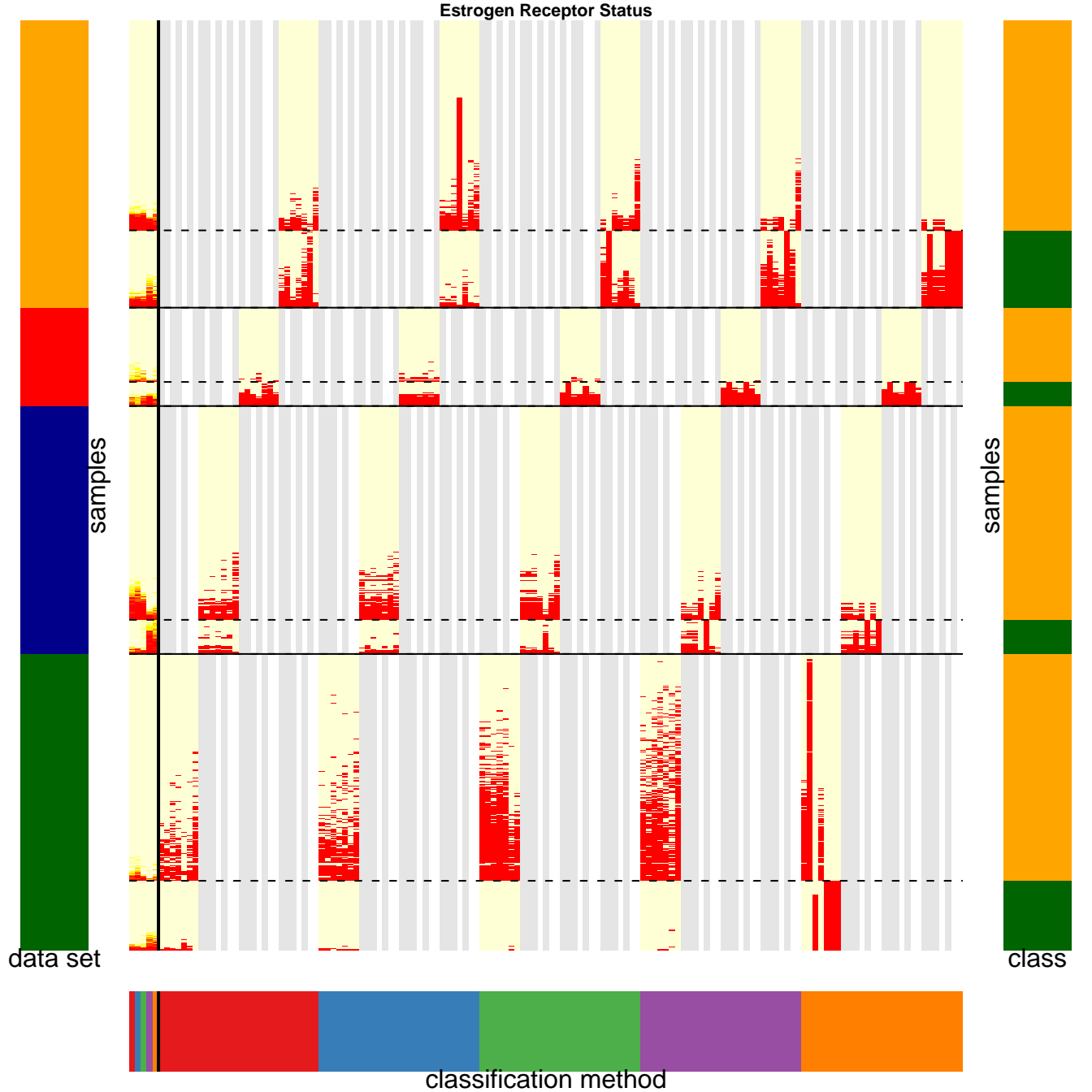

Supplement: Additional file 3 — Classification results across studies for estrogen receptor status. The figure summarizes all classification results for estrogen receptor status. Samples correspond to rows and methods to columns. The estimates of the cross-validation approach are shown on the left separated by a vertical line from the results of the classification across studies on the right. For the latter the samples of the studies used for the training are marked in gray and the ones not used are shown in white. The cross-validation approach was run separately for each study. Misclassified samples are labelled in red and correctly classified ones in light yellow. The error estimates of the repeated cross-validation have been mapped to the range from red to light yellow. Samples are ordered by study, class, their average misclassification rate in the cross-validation and classification across studies. The color code at the bottom indicates the method (red = DV, blue = kTSP, green = PAM, purple = RF, orange = SVM), at the left the study (green = 1, blue = 2, red = 3, orange = 4), at the right the class (green = ER-, orange = ER+). [file 1471-2105-10-453-S3.PDF]

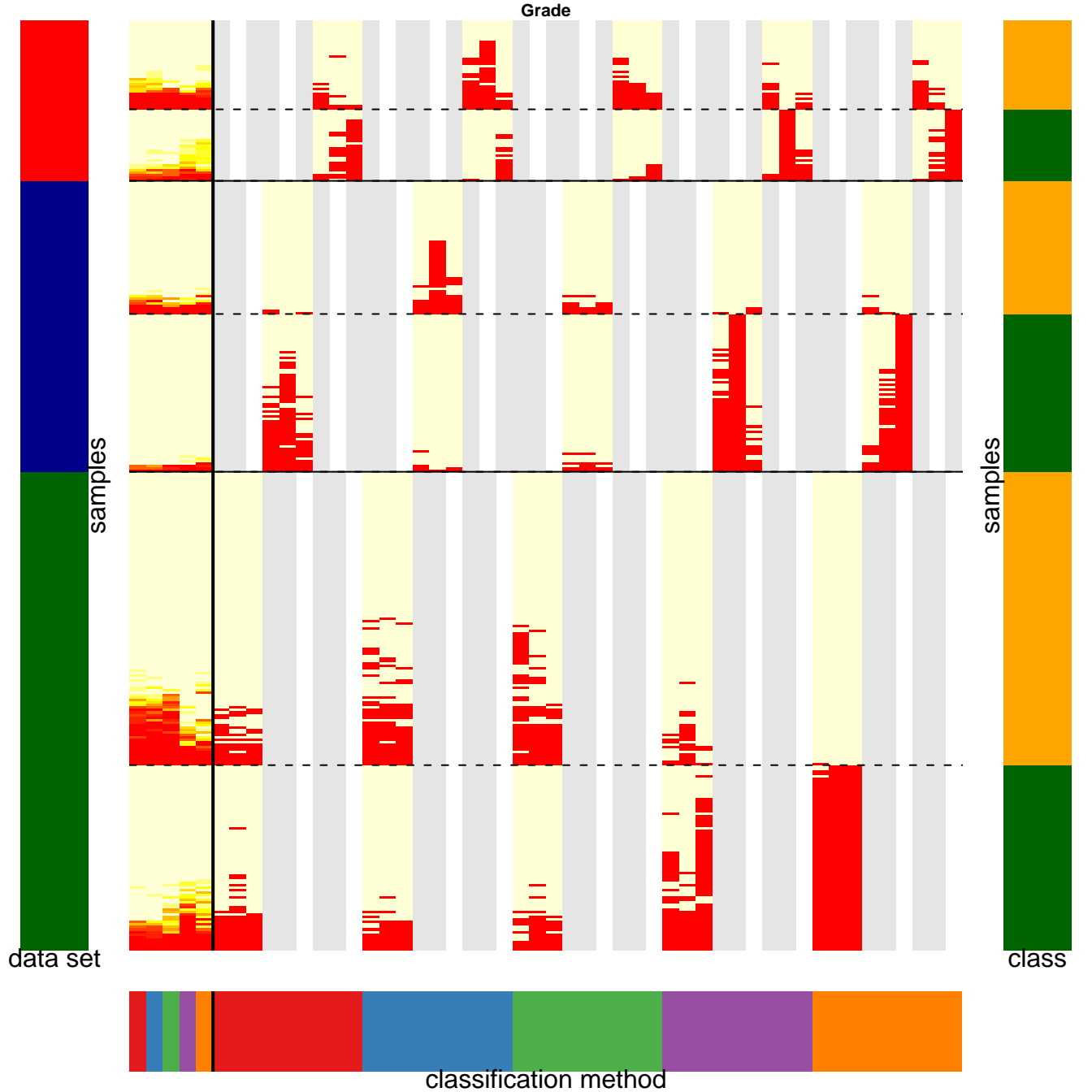

Supplement: Additional file 4 — Classification results across studies for histological grade. The figure summarizes all classification results for the histological grade. Samples correspond to rows and methods to columns. The estimates of the cross-validation approach are shown on the left separated by a vertical line from the results of the classification across studies on the right. For the latter the samples of the studies used for the training are marked in gray and the ones not used are shown in white. The cross-validation approach was run separately for each study. Misclassified samples are labelled in red and correctly classified ones in light yellow. The error estimates of the repeated cross-validation have been mapped to the range from red to light yellow. Samples are ordered by study, class, their average misclassification rate in the cross-validation and classification across studies. The color code at the bottom indicates the method (red = DV, blue = kTSP, green = PAM, purple = RF, orange = SVM), at the left the study (green = 1, blue = 2, red = 3), at the right the histological grade (green = G1, orange = G3). Study 4 is not included since the histological grade of the samples was not available. [file 1471-2105-10-453-S4.PDF]
