# Supplementary material for: Classification across gene expression microarray studies
Source: BMC Bioinformatics. 2009 Dec 30;10:453. doi: 10.1186/1471-2105-10-453 (PMC2811711; doi:10.1186/1471-2105-10-453)
Supplement: Additional file 5 — Gene signatures. Gene signatures for the prediction of estrogen receptor status and histological grade derived from the complete data set. [file 1471-2105-10-453-S5.PDF]

Supplemental Table 1: The gene signature of the DV and kTSP classifier for the prediction of estrogen receptor status derived from the complete data set.

Gene Symbols
ACADSB, ACTR3, ADAMDEC1, ADM, AGR2, ASS, C10orf116, CA12, CD53, CDH3, CDK2AP1, CP, CTSC, DNAJC12, DNALI1, EIF3S6, ERBB4, EVL, FLJ20152, FOXA1, FOXC1, GATA3, GBP1, JMJD2B, LASS2, NAT1, PADI2, PDHA1, PDIA6, PFDN5, PFKP, PROM1, PSMB2, PTP4A2, RARRES1, RHOB, S100A8, S100A9, SCUBE2, SERPINA5, SLC39A6, SOX11, SSR4, STC2, TFF1, TFF3, TPBG, UQCRH, XBP1, YBX1

Supplemental Table 2: The gene signature of the PAM classifier for the prediction of estrogen receptor status derived from the complete data set.

Gene Symbols
ACADSB, ADAMDEC1, ADM, AGR2, AGTR1, AKR7A3, AREG, ASS, ATP1B3, BLVRA, C10orf116, C1orf21, C4B, CA12, CALML5, CCL5, CCNB2, CD300A, CDH3, CHAD, CHI3L1, CITED1, CP, CPA3, CPB1, CRABP1, CTSC, CYP2B6, CYP4B1, DHRS2, DNAJC12, DNALI1, DUSP4, EEF1A2, ENO1, ERBB3, ERBB4, ESR1, EVL, FABP5, FABP7, FLJ20152, FOXA1, FOXC1, GABRP, GATA3, GBP1, GREB1, GSTM3, HDAC11, IGFBP2, IGFBP4, INDO, JMJD2B, KCMF1, KIAA1467, KRT7, LRIG1, LRP2, LTB, MAGED2, MAOA, MIA, MMP1, MUC1, NAT1, NOVA1, NPY1R, NRIP1, PADI2, PCSK6, PDHA1, PDZK1, PFKP, PGR, PIP, POU2AF1, PRAME, PROM1, PSD3, QDPR, RAB27B, RARRES1, RIS1, S100A8, S100A9, SCGB1D2, SCNN1A, SCUBE2, SEC14L2, SERPINA5, SERPINB5, SFRP1, SGK3, SH3BGRL, SIAH2, SLC16A6, SLC27A2, SOX11, STC2, TBX3, TFF1, TFF3, THBS4, THRAP2, TNNT1, TPBG, TSPAN1, UGCG, UNG2, VAV3, WARS, XBP1, YBX1, ZIC1

Supplemental Table 3: The gene signature of the PAM classifier for the prediction of histological grade derived from the complete data set.

Gene Symbols
ABAT, ACADSB, ADAMDEC1, AGTR1, ASPN, C1orf78, C20orf28, C4B, CA12, CCNB2, CCNG2, CENPA, CHAD, CILP, CXCL11, DACH1, DNAJC12, DNALI1, EGR3, ERBB4, FMOD, FOS, FOXA1, GATA3, GJA1, GLI3, GSTM3, HMGB3, HOXB2, IGFBP4, KIF13B, KIF2C, LAMP3, LRIG1, LRP2, LRRC17, MAOA, MATN3, MMP1, NAT1, NPY1R, PCSK6, PGM5, PIP, PLAT, PLIN, PSD3, QDPR, RUTBC1, SCUBE2, SFRP4, SOX11, STC2, TGFBR3, THBS4, TIMP4, UBE2C, VAV3, WISP2, ZBTB16
