# Supplementary material for: Classification across gene expression microarray studies
Source: BMC Bioinformatics. 2009 Dec 30;10:453. doi: 10.1186/1471-2105-10-453 (PMC2811711; doi:10.1186/1471-2105-10-453)
Supplement: Additional file 6 — Description of DV. Algorithmic description of DV. [file 1471-2105-10-453-S6.PDF]

Let the measurements be given as a genes $\times$ samples matrix (q_{gs}), where q_{gs} denote the relative rank (the quantile) of the expression value of gene g in sample s among all expression values of sample s (i.e. q_{gs} is the rank of gene g, divided by the number of genes in sample s for which measurements are avaliable). Each sample s belongs to one class C_l of size $|C_l|$ $(l = 1, 2)$. The essential building block of the method is the comparison of genes and given by the function h:

$$h(g_i, g_j) = \begin{cases} 1, & g_i < g_j \\ -1, & otherwise. \end{cases}$$

The absolute score $|\Delta_{ij}|$ of a gene pair (i, j)

$$\Delta_{ij} = \sum_{l=1,2} (-1)^l \sum_{s \in C_l} \frac{h(g_{is}, g_{js})}{|C_l|}$$

is used to rank the gene pairs. Then, starting with the top scoring gene pair and following their rank ordering, gene pairs are successively evaluated for inclusion in the classifier. The gene pair $(\hat{i}, \hat{j})$ is selected for the classifier if the follwing two criteria are met: $\sum_i I(i, \hat{j}) \leq F$, $\sum_j I(\hat{i}, j) \leq F$, where $I(i, j)$ denotes a indicator function, which is 1 if the pair (i, j) belongs to the already selected pairs and 0 otherwise. F is a parameter of the classificator. A gene participates in at most F pairwise comparisons of our final selection. The criteria aim to balance and control the amount of cross-linking between the genes. Then the class of a new sample s^* is determined as follows:

$$class(s^*) = f(\sum_{i,j} sign(\Delta_{ij}) h(g_{is^*}, g_{js^*}) I(i, j)) \quad ,$$

where the function I indicates the final set of selected gene pairs and the function f computes the class membership based on the their ‘votes‘ as follows:

$$f(x) = \begin{cases} 1, & x \leq 0 \\ 2, & x > 0 \end{cases} .$$

Here, we only consider all pairs of genes in the set of two times k genes which have been selected by the kTSP classifier. thus the kTSP classifier itself is a special case of DV for the parameter choice $F = 1$. A tie in the votes does not cause a problem, since we can simply choose any of the two alternatives as done above. A tie situation means that the posterior odds for C_1 versus C_2 is 1, and we may use any decision rule, which always ends up with a 50% chance of guessing the right class. When aiming to build a reasonable classifier one should account for the counting based score Δ_{ij} and ensure that the number of samples exceeds at least 20 in total. On the other hand, the counting based score Δ_{ij} allows to easily integrate any further microarray study in an already existing classifier rule and thus to subsequently extend the sample size.
